# Platelet-Rich Plasma Promotes the Expansion of Human Myoblasts and Favors the *In Vitro* Generation of Human Muscle Reserve Cells in a Deeper State of Quiescence

**DOI:** 10.1007/s12015-024-10760-0

**Published:** 2024-07-13

**Authors:** Axel Tollance, Alexandre Prola, Diego Michel, Axelle Bouche, Antoine Turzi, Didier Hannouche, Sarah Berndt, Thomas Laumonier

**Affiliations:** 1 Department of Orthopedic Surgery, Geneva University Hospitals & Faculty of Medicine, Geneva, Switzerland; 2 Department of Cell Physiology and Metabolism, Faculty of Medicine, Geneva, Switzerland; 3 Regen Lab SA, 1052 Le Mont-Sur-Lausanne, Switzerland

**Keywords:** Human myoblasts, Muscle reserve cell, Platelet-rich plasma, Hyaluronic acid, Pax7, Quiescence

## Abstract

Stem cell therapy holds significant potential for skeletal muscle repair, with in vitro-generated human muscle reserve cells (MuRCs) emerging as a source of quiescent myogenic stem cells that can be injected to enhance muscle regeneration. However, the clinical translation of such therapies is hampered by the need for fetal bovine serum (FBS) during the in vitro generation of human MuRCs. This study aimed to determine whether fresh allogeneic human platelet-rich plasma (PRP) combined or not with hyaluronic acid (PRP-HA) could effectively replace xenogeneic FBS for the ex vivo expansion and differentiation of human primary myoblasts. Cells were cultured in media supplemented with either PRP or PRP-HA and their proliferation rate, cytotoxicity and myogenic differentiation potential were compared with those cultured in media supplemented with FBS. The results showed similar proliferation rates among human myoblasts cultured in PRP, PRP-HA or FBS supplemented media, with no cytotoxic effects. Human myoblasts cultured in PRP or PRP-HA showed reduced fusion ability upon differentiation. Nevertheless, we also observed that human MuRCs generated from PRP or PRP-HA myogenic cultures, exhibited increased Pax7 expression and delayed re-entry into the cell cycle upon reactivation, indicating a deeper quiescent state of human MuRCs. These results suggest that allogeneic human PRP effectively replaces FBS for the ex vivo expansion and differentiation of human myoblasts and favors the in vitro generation of Pax7^High^ human MuRCs, with important implications for the advancement of stem cell-based muscle repair strategies.

**Graphical Abstract:**

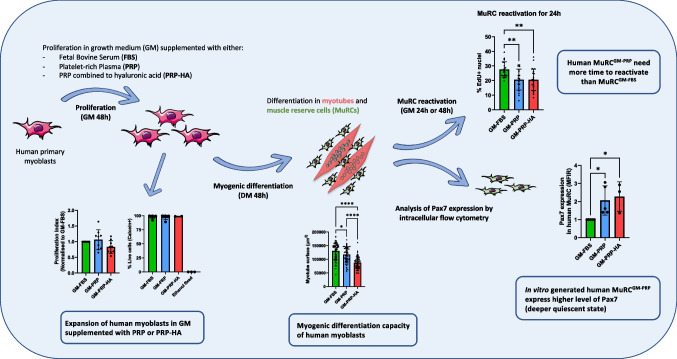

**Supplementary Information:**

The online version contains supplementary material available at 10.1007/s12015-024-10760-0.

## Introduction

Muscle stem cells (MuSCs), located between the muscle fiber and the basal lamina, are quiescent myogenic stem cells known to be essential for postnatal muscle growth and skeletal muscle regeneration [1, 2]. Following muscle injury, MuSCs are activated and re-enter the cell cycle to proliferate as myoblasts. Myoblasts can then either differentiate to repair damaged muscle fibers or return to a quiescent state to replenish the MuSC pool [3]. In congenital muscle diseases such as Duchenne muscular dystrophy (DMD), MuSCs do not function as healthy one and their impairment contributes to disease progression [4]. Although MuSC-based therapy has emerged as a promising therapeutic target for muscle repair [5, 6], progress in this field awaits a better definition of the type of cells to use and how to increase their number and myogenic capacity in diseased muscles [7]. We have recently shown that human muscle reserve cells (MuRCs) share many of the characteristics of quiescent MuSCs and are heterogeneous for Pax7 expression, with a Pax7^High^ subpopulation in a deeper quiescent state [8]. We have also shown that human MuRCs survive and participate in muscle regeneration after injection into the injured muscles of immunodeficient mice [8, 9]. Furthermore, quiescent MuRCs can be generated in large numbers and display immunosuppressive capacities in vitro [10], making them a promising source of cells for cell therapy purposes.

Fetal bovine serum (FBS) has traditionally been used as a source of nutrients and growth factors for human myoblast expansion, raising risks of pathogenicity, cell immunogenicity [11–13] and ethical concerns [14]. These issues highlight the need to find alternatives to the use of FBS for the in vitro expansion of human cells. Serum-free media or a variety of human blood derivatives have already been tested with different types of human cells, including platelet-rich plasma (PRP) of both autologous and allogeneic origin [15–20]. PRP is described as a platelet-rich plasma, without erythrocytes and with varying concentrations of white blood cells, depending on the protocol used to prepare it [21, 22]. Two main PRP preparations are commonly used, a leukocyte-rich platelet-rich plasma (LR-PRP) and a leukocyte-poor platelet-rich plasma (LP-PRP) [23, 24], in an inactivated or activated states [25, 26]. The nomenclature surrounding PRP is also extended to include platelet lysate, a concentrated heat-inactivated plasma rich in growth factors [21, 22, 27] or platelet releasate, a supernatant containing growth factors released from activated platelets [28, 29]. The term PRP has also been used to describe a freshly isolated PRP containing non-activated platelets that gradually release growth factors over time in culture [30, 31]. We have recently shown that autologous non-activated PRP improves the in vitro expansion of human fibroblasts as compared to xenogeneic FBS [32, 33]. Few studies have evaluated the use of different PRP preparations for myogenic cell cultures. The proliferation of rat myoblasts or murine myoblasts was shown to be enhanced when cells were expanded in media supplemented with either LR-PRP, LP-PRP [34, 35] or platelet releasate [29, 36]. It was also shown that autologous plasma lysate, serum or PRP, favors the expansion of human myogenic cells, in particular by combining PRP with decorin, a TGF-β inhibitor [37–40]. Despite discrepancies regarding the effect of PRP or its derivatives on myogenic proliferation and/or differentiation, it appears that PRP is a viable alternative to xenogeneic sera [38, 39, 41]. Nevertheless, although autologous PRP is ideal for avoiding potential immune/inflammatory reactions, its use is limited by the difficulty of controlling the quality of individual PRP preparation and the difficulties of obtaining large amounts of autologous PRP [42]. Moreover, hyaluronic acid (HA) is an important component of the extracellular matrix (ECM) in skeletal muscle [43], which has been shown to play an important role in regulating MuSC behavior during muscle regeneration, as well as regulating cell–cell interactions and modulating the local concentration of soluble factors in the microenvironment [44, 45].

Therefore, in this study, we investigated two human blood derivatives allogeneic non-activated human PRP (PRP) or allogeneic non-activated human PRP combined with hyaluronic acid (PRP-HA), used as FBS substitutes for the in vitro expansion of primary human myoblasts prior to their myogenic differentiation in myotubes and in MuRCs. We evaluated the in vitro proliferation rate, the expression of various inflammatory cell surface markers, their myogenic differentiation capacity and the level of Pax7 expression in human MuRCs. We have shown that PRP or PRP-HA can efficiently replace xenogeneic FBS in the in vitro expansion of human myoblasts, culture conditions that do not alter the expression of inflammatory cell surface markers. We also showed that human myoblasts, expanded in growth medium containing either PRP or PRP-HA, generated a higher percentage of Pax7^High^ MuRCs as compared to myoblasts expanded in growth medium containing FBS. Our results strongly suggest that human allogeneic non-activated PRP or PRP-HA are efficient and suitable alternatives to FBS for the expansion of human primary myoblasts and for the generation of human MuRCs expressing higher levels of Pax7.

## Materials & Methods

### Cell Culture

Human primary myoblasts were purified from semitendinosus muscle samples following orthopedic surgery (surgical waste) of patients without known muscular diseases. The isolation of human primary myoblasts was performed as previously described [46]. Briefly, muscles were mechanically and enzymatically (trypsin–EDTA, Thermo Fisher, St. Louis, MO, USA) dissociated to keep only mononucleated cells. Cells were then amplified and sorted by flow cytometry to isolate a pure population of human myoblasts (CD56^+^ / CD82^+^ / CD146^+^ cells).

Myoblasts were cultured in a growth medium (GM) containing either 15% of fetal bovine serum (FBS, Gibco), 15% of human PRP (Regen BCT, RegenLab, Switzerland) or 15% of human PRP-HA (Regen BCT-HA, RegenLab, Switzerland). Heparin (heparin-NA,2 IU/ml, Braun) was added to all FBS- and PRP-supplemented media to prevent the gelation of the medium. At 80% confluence, human myoblast differentiation was induced by replacing GM-FBS, GM-PRP or GM-PRP-HA with differentiation medium (DM) for 48 h. The composition of GM and DM media has been previously described [47]. After 48 h in DM, human MuRCs were separated from myotubes after short trypsinization and filtration using 20 µm pre-separation filters (Miltenyi Biotec, Bergisch Gladbach, Germany). Brightfield pictures of human myoblast cultures were taken on a Nikon Eclipse Ts2 equipped with a 10X/0.25 objective.

Myoblasts and MuRCs numbers were determined using the automatic cell counter CellDrop BF (DeNovix, Wilmington, USA).

### PRP Preparation

Human platelet-rich plasma PRP was prepared freshly using the Regen BCT tube device (Regen BCT, RegenLab, Switzerland) and human PRP-HA was prepared freshly using the Regen BCT-HA (Regen BCT-HA, RegenLab, Switzerland) device [48]. All BCT and BCT-HA tubes contained a thixotropic gel and a reversible anticoagulant (sodium citrate). The BCT-HA tube contained 2 ml of cross-linked hyaluronic acid (20 mg/ml). After centrifugation at 1500 g for 5 min, standardized PRP or PRP-HA preparations, defined as leucocyte poor PRP (LP-PRP) [48], were stored at room temperature for up to 10 days.

### Immunofluorescence

Cells were fixed in PBS 4% paraformaldehyde, permeabilized, and then blocked with PBS containing 0.3% Triton X-100 and 5% goat serum. For immunofluorescence, an overnight incubation was carried out at 4 °C using the following primary antibodies: mouse anti α-actinin (1:500, A7811, Sigma, Taufkirchen, Germany) and rabbit anti MEF2C (1:500, 5030S, Cell Signaling, Danvers, MA, USA). After 3 washing in PBS, cells were incubated at room temperature (RT) for 75 min with the following secondary antibodies: Alexa Fluor^®^ 488-conjugated goat anti-Mouse IgG (1:1000, A11029, ThermoFisher, Carlsbad, CA, USA) and Alexa Fluor^®^ 546-conjugated goat anti-Rabbit IgG (1:1000, A11030, ThermoFisher).

For the EdU assay, human MuRCs were reactivated for 24 h or 48 h in GM-FBS containing Edu. Cells were then, fixed, permeabilized and stained with an anti-Edu Alexa Fluor^®^ 647 (Click-iT™ Plus EdU Kit, C10640, ThermoFisher). Nuclei were labeled using the ProLong^®^ Gold antifade reagent containing DAPI (P36931, ThermoFisher). Images were captured using a Widefield AxioImager M2 microscope (ZEISS, Germany) equipped with an EC Plan-Apochromat 10X/0.8 objective. Analysis of the immunofluorescence images was performed using the software QuPath [49] under systematic user supervision and with a manual correction to minimize cell counting errors.

### Western Blot

Total protein extract was obtained by harvesting human myoblasts or MuRCs after trypsinization and filtration. Cells were centrifuged, and cell pellets were rinsed twice with PBS, followed by immediate lysis in CHAPS 1%. The cell lysates were then centrifuged for 5 min at 13,000 rpm, and the protein content of the supernatant was determined using a BCA kit assay (Pierce™ BCA Protein Assay Kits Cat. No.23225). Total proteins (10 µg) were separated on a 10% SDS–polyacrylamide gel and transferred to nitrocellulose membranes (Macherey–Nagel, Düren, Germany). The membranes were saturated in TTBS (Tween/Tris-buffered saline; 0.1% Tween 20, 20 mmol/l Tris–HCl (pH 7.5), and 137 mmol/l NaCl) containing 1% PVA. Next, the membranes were incubated with the mouse anti-human Pax7 (1:300, DSHB) and mouse anti-α-tubulin (1:6000, Sigma-Aldrich) primary antibodies, which were diluted in TTBS-3% BSA and kept overnight at 4 °C. Membranes were washed three times with TTBS for 10 min and subsequently incubated for 1 h with HRP-conjugated goat anti-mouse (1:10000, BioRad, Hercules, CA, USA). Blots were revealed using ECL reagents (Perkin-Elmer) and mxECL Imager (ThermoFisher). The level of protein expression was quantified by performing image analysis using FiJi software (ImageJ).

### Flow Cytometry

#### Cell surface markers staining

Human myoblasts were cultured for 48 h in the following culture medium: GM-FBS, GM-FBS-IFNγ, GM-PRP or GM-PRP-HA. For the GM-FBS-IFNγ, cells were incubated in GM-FBS containing 500 units/ml of human IFNγ (Peprotec, Cat. N°. 300–02) for 48 h. Cells were then trypsinized, washed in PBS and stained with the following antibodies for 30 min at 4 °C: FITC mouse Anti-Human HLA-ABC (BD Biosciences Cat. No.555552, 20 µl/1 M cells), FITC mouse anti-human HLA-DR (BD Biosciences Cat. No. 555811, 20 µl/1 M cells), PE mouse anti-human CD54 (BD Biosciences Cat. No. 555511, 20 µl/1 M cells), Alexa Fluor^®^ 488 mouse anti-human CD56 (BD Biosciences Cat. No. 557699, 5 µl/1 M cells) or PE mouse anti-human CD56 (BD Biosciences Cat. No. 555516, 20 µl/1 M cells). Negative controls were treated with an equivalent quantity of FITC- and PE-labeled isotype-matched antibodies. Cells were then washed twice with PBS and suspended in 400 μl of PBS until acquisition. Acquisitions were performed on a Beckman Coulter Cytoflex.

#### Intracellular Pax7 staining

Human MuRCs were trypsinized, washed twice in PBS and stained with Fixable Viability Stain (FVS; 1:1000, BD Biosciences, Cat. No. 565388) for 10 min at RT to target only live cells. Cells were then washed twice with PBS and fixed/permeabilized with a Transcription Factor Buffer Set (BD Biosciences, Cat. No. 562574). Cells were then incubated with Human TruStain FcX™ (BioLegend, cat#422,302, 5 µl/1 M cells) for 5 min at RT and stained with the following antibodies for 40 min at 4 °C: Alexa Fluor^®^ 488 mouse anti-human CD56 (BD Biosciences Cat. No. 557699), Alexa Fluor^®^ 647 mouse anti-human Pax3/7 (Santa Cruz Biotechnology, Dallas, TX, USA, Cat#sc-365843). Negative controls were treated with an equivalent amount of FITC-labeled and Alexa Fluor^®^ 647-labeled isotype-matched antibodies. Cells were then washed three times with Perm/Wash buffer and resuspended in 400 μl PBS until acquisition. The acquisition was performed using the Beckman Coulter Cytoflex.

### Live Dead Staining

Human myoblasts were cultured in GM-FBS, GM-PRP or GM-PRP-HA for 48 h and their viability was evaluated using a LIVE/DEAD^®^ Viability/Cytotoxicity Kit (Molecular Probes) following supplier protocol. Briefly, live cells were detected based on their incorporation of Calcein-AM, while dead cells were positive for Ethidium homodimer-1 due to their damaged membrane. Human myoblasts permeabilized with ethanol 70% for 20 min were used as a positive control for dead cells. The acquisition was performed using the Beckman Coulter Cytoflex.

### Statistics

Data are presented as mean ± SD, and statistical differences were determined using the statistical test specified in the figure legends, with * p < 0.05, ** p < 0.01, ***p < 0.001, and **** p < 0.0001. For the comparison of two populations, a t-test or Mann–Whitney test was used depending on the normality of the dataset. For the comparison of more than two populations, either ANOVA or the Kruskal–Wallis test was used, depending on the normality of the dataset.

## Results

### The Ability of Human Primary Myoblasts to Expand *In Vitro* is Similar when Grown on Media Supplemented with either Xenogeneic FBS, Allogeneic PRP or Allogeneic PRP-HA

**Fig. 1:**
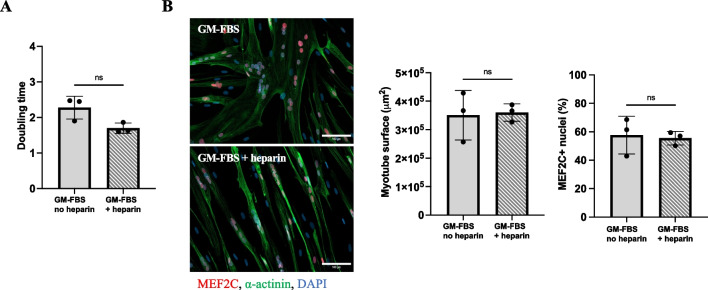
Heparin supplementation has no effect on the proliferation and differentiation of human myoblasts. (A) Human myoblasts were expanded for 48 h in GM-FBS containing heparin (2 UI/ml) and counted. The population doubling level (PDL) was determined using the following formula: PDL = 3.32 (log (total viable cells at harvest/total viable cells at seed). (B) Myoblasts expanded in either GM-FBS or GM-FBS-heparin, were then differentiated for 48 h in DM. Representative images of differentiated myogenic culture after 48 h in DM, stained for MEF2C (orange), α-actinin (green) and DAPI (blue), (Scale bar = 100 µm). Quantification of myotube surface area (N = 3, mean ± SD, Mann–Whitney test) and quantification of the percentage of MEF2C positive nuclei (N = 3, mean ± SD, unpaired t-test)

**Fig. 2:**
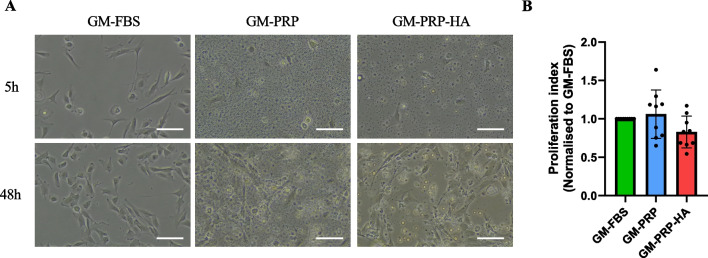
Allogeneic PRP or PRP-HA promotes human myoblast proliferation. (A) Representative images of human myoblasts expanded in growth medium (GM) containing 15% of FBS (GM-FBS) or 15% of allogeneic PRP (GM-PRP) or 15% of allogeneic PRP-HA (GM-PRP-HA) at 5 h or 48 h after plating (Scale bar: 100 µm). (B) Proliferation index of human myoblasts, normalized to GM-FBS, after 48 h in either GM-FBS, GM-PRP or GM-PRP-HA (N =9, mean ± SD)

Primary human myoblasts were seeded at 3500 cells/cm^2^ and cultured for 48 h in a growth medium supplemented with either 15% of FBS (GM-FBS), 15% of PRP (GM-PRP), or 15% of PRP-HA (GM-PRP-HA). All culture media were supplemented with 2 IU/ml of heparin. We first observed that the addition of 2 IU/ml of heparin in GM did not alter the growth kinetics of human myoblasts (Fig. 1A). Myoblasts expanded in either GM-FBS or GM-FBS-heparin were then switched to differentiation medium (DM) for 48 h. We noticed that the myotube surface area and the percentage of MEF2C positive nuclei was similar in both conditions (Fig. 1B). We then assessed the effect of allogeneic PRP on human myobalsts. Five hours after plating, we observed that myoblasts cultured in either GM-PRP or GM-PRP-HA adhered to the culture plates more slowly than myoblasts cultured in GM-FBS (Fig. 2A). After 48 h in growth medium, the proliferation index was not significantly different in all conditions tested. Proliferation index was 1.06 ± 0.32 and 0.82 ± 0.21 (mean ± SD) for human myoblasts cultured in GM-PRP and GM-PRP-HA, respectively, compared to 1 for those cultured in GM-FBS (Fig. 2B**)**.

### GM-PRP or GM-PRP-HA did not Alter the Expression of Inflammatory Cell Surface Markers

**Fig. 3:**
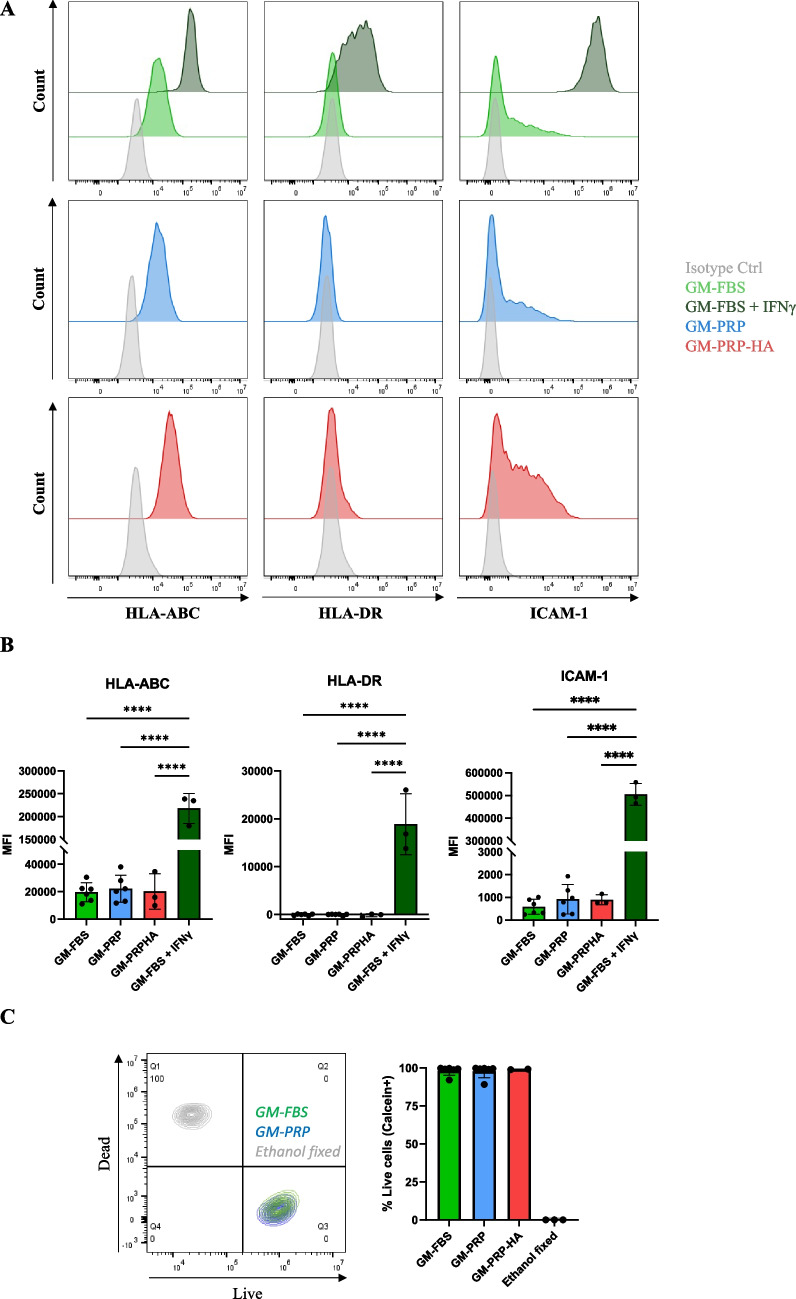
Culture with allogeneic PRP does not induce upregulation of HLA-ABC, HLA-DR or ICAM-1 and does not increase mortality in human myoblasts. Human myoblasts were expanded for 48 h in growth medium (GM) containing 15% of FBS (GM-FBS), 15% of allogeneic PRP (GM-PRP), 15% of allogeneic PRP-HA (GM-PRP-HA) or 15% of FBS supplemented with interferon γ (GM-FBS + IFNγ) and analyzed by flow cytometry for the expression of HLA-ABC, HLA-DR and CD54. (A) Representative examples of flow cytometry results and (B) quantification of the median of fluorescence intensity (MFI, N = 3–6, mean ± SD). (C) Live/dead assay of human myoblasts after 48 h expansion in GM-FBS or GM-PRP. Ethanol-treated myoblasts were used as a positive control. Representative data and histogram of the percentage of calcein + live cells (N = 2–4, mean ± SD)

We investigated whether human myoblasts cultured in GM-FBS, GM-PRP or GM-PRP-HA for 48 h share a similar phenotype in terms of major histocompatibility complex (MHC) class I (HLA-ABC), MHC class II (HLA-DR) and intracellular adhesion molecule 1 (ICAM-1) expression, and compared to myoblasts cultured under inflammatory conditions (GM-FBS + IFNγ) [50]. Flow cytometry analysis showed that all human myoblasts cultured in GM-FBS express the cell surface marker HLA-ABC. Human myoblasts were also positive for ICAM-1 expression but were negative for HLA-DR (Fig. 3A). We then compared the median fluorescence intensity (MFI) for each cell surface marker analyzed. Expansion of human myoblasts in GM-PRP or GM-PRP-HA for 48 h did not significantly affect the expression of HLA-ABC, HLA-DR or ICAM-1, as compared to GM-FBS (Fig. 3B). On the contrary, HLA-ABC, HLA-DR and ICAM-1 expression were significantly up-regulated in myoblasts cultured in GM-FBS + IFNγ for 48 h (Fig. 3B). We also evaluated the viability of human myoblasts after expansion in GM-FBS, GM-PRP and GM-PRP-HA media for 48 h. We observed that allogeneic PRP did not induce any significant cell toxicity in vitro, with less than 2% of dead cells in all conditions tested as compared to 100% of dead cells in the ethanol-fixed group (Fig. 3C).

### Significant Inhibitory Effect of Allogeneic GM-PRP or GM-PRP-HA on Human Myogenic Differentiation

**Fig. 4:**
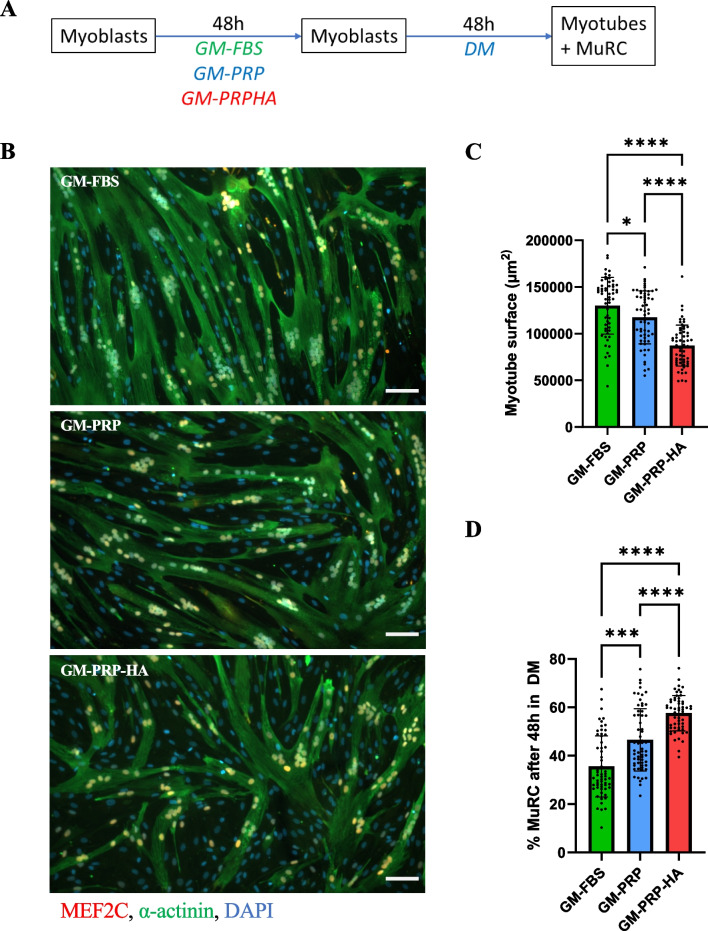
Human myoblasts expanded in GM-PRP or GM-PRP-HA display reduced myogenic differentiation capacities. (A) Myoblasts were cultured in either GM-FBS, GM-PRP or GM-PRP-HA for 48 h, switched to DM for 48 h and stained for MEF2C (orange), α-actinin (green) and DAPI (blue). (B) Representative images of human myotubes, defined as α-actinin + /MEF2C + /DAPI + and human MuRCs defined as α-actinin-/MEF2C-/DAPI + (Scale bar = 100 µm). (C) Quantification of myotube surface area (N = 6, mean ± SD, Mann–Whitney test and Dunn’s multiple comparisons test) and (D) quantification of the percentage of human MuRCs (N = 6, means ± SD, Kruskal–Wallis test and Dunn’s multiple comparisons test). *p < 0.05, **p < 0.01, ***p < 0.001, ****p < 0.0001

Human myoblasts were cultured to 80% confluence in either GM-FBS, GM-PRP or GM-PRP-HA and then switched to differentiation medium (DM) for 48 h. Cells were then fixed and stained for α-actinin and MEF2C (Fig. 4A). We observed that human myoblasts expanded in GM-FBS, GM-PRP or GM-PRP-HA can differentiate and form α-actinin-positive myotubes in vitro (Fig. 4B). We also found that the surface coverage of myotubes (α-actinin staining in green and nuclei positive for MEF2C in orange) was significantly reduced in GM-PRP or GM-PRP-HA conditions compared to GM-FBS with a decrease of 9.7% and 32.8%, respectively (average 1.3 × 10^5^ ± 3.1 × 10^4^ µm^2^ for GM-FBS vs. 1.2 × 10^5^ ± 2.8 × 10^4^ µm^2^ for GM-PRP and 8.7 × 10^4^ ± 2.2 × 10^4^ µm^2^ for GM-PRP-HA, mean ± SD, Fig. 4C). Conversely, the proportion of cells that escape terminal differentiation known as MuRCs, was significantly increased in the GM-PRP and GM-PRP-HA conditions (35.6 ± 12.7% for GM-FBS vs. 46.6 ± 12.9% for GM-PRP and 57.6 ± 7.39% for GM-PRP-HA, mean ± SD, Fig. 4D).

### Human Myoblasts Expanded in GM-PRP or GM-PRP-HA Favor the *In Vitro* Generation of Pax7^High^ Human MuRCs

**Fig. 5:**
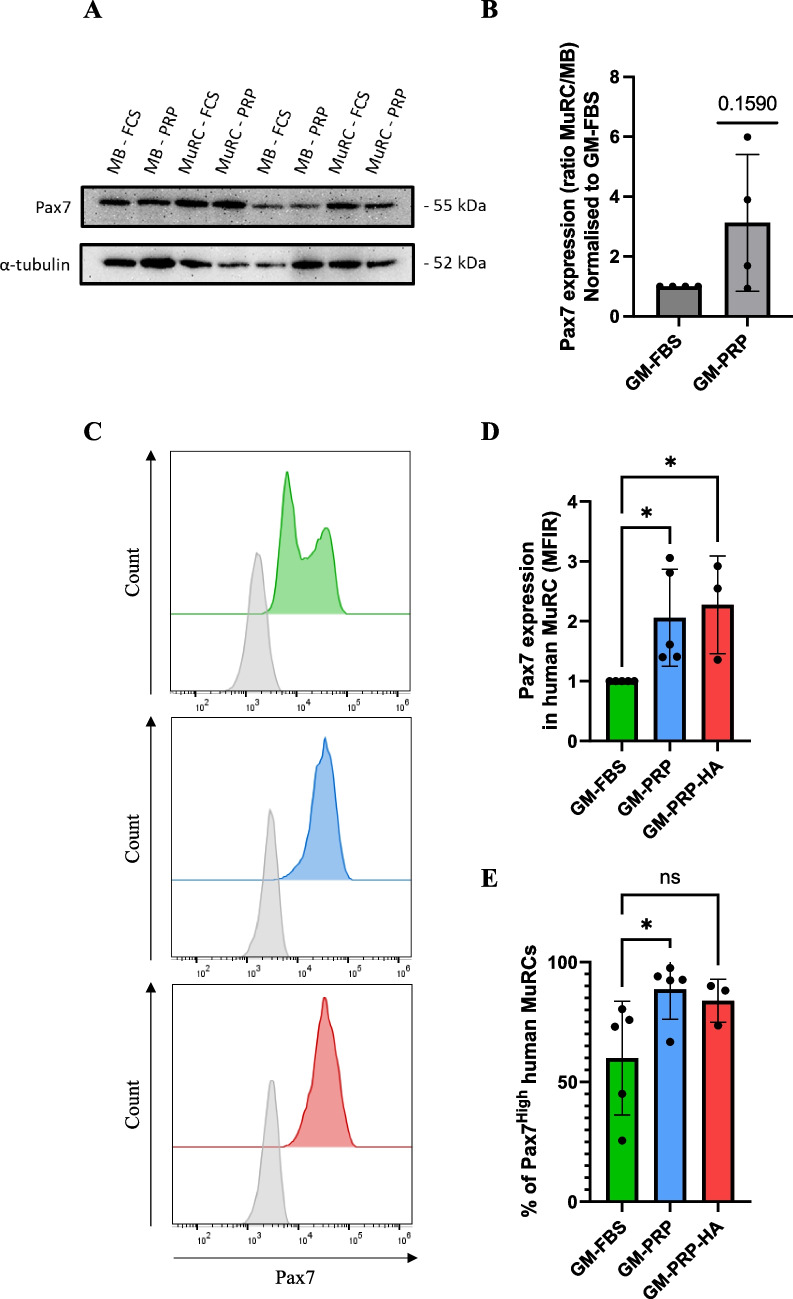
Human MuRCs generated in vitro from PRP-expanded myoblasts express higher levels of Pax7. Human myoblasts were expanded in GM-FBS, GM-PRP or GM-PRP-HA for 48 h and then switched to DM for 48 h. Human MuRCs were isolated and analyzed for Pax7 expression by Western blot and flow cytometry. (A) Representative Western blot for Pax7 and α-tubulin expression. (B) Quantification of Pax7 expression (ratio MuRC to myoblasts), normalized to Pax7 expression in GM-FBS. (C) Representative histogram of flow cytometry analysis for Pax7 expression in MuRCs generated from myoblasts expanded in either GM-FBS, GM-PRP or GM-PRP-HA. In (D) and (E), histograms showing the median fluorescence intensity ratio (MFIR) for Pax7 expression compared to GM-FBS (N = 5 for GM-PRP and N = 3 for GM-PRP-HA, mean ± SD, unpaired t-test, *p < 0.05)

After 48 h in DM, we observed an increase in the percentage of human MuRCs after differentiation of myoblasts expanded in either GM-PRP or GM-PRP-HA as compared to GM-FBS (Fig. 4D). We then further characterize the myogenic status of human MuRCs by evaluating the level of Pax7 expression by Western blot and by flow cytometry. By Western blot, we observed an increase of Pax7 expression in MuRC generated from GM-PRP myoblasts (MuRC-PRP) as compared to MuRC generated from GM-FBS myoblasts (MuRC-FBS). When normalised to α-tubulin and compared to myoblasts expanded in GM-FBS (MB-FBS), we observed a 3.1-fold increase in Pax7 expression in MuRC-PRP compared to MuRC-FBS (*p* = 0.159, Fig. 5A and B). Using flow cytometry, we observed a twofold increase in Pax7 expression in human MuRCs generated from GM-PRP myoblasts and a 2.3-fold increase in human MuRCs generated from GM-PRP-HA myoblasts compared to human MuRCs generated from GM-FBS myoblasts **(**Fig. 5C and D). Consistent with the level of Pax7 expression, we also found that the proportion of Pax7^High^ MuRCs was significantly increased in GM-PRP or GM-PRP-HA, with 88 ± 12.4% and 83 ± 9.0% of Pax7^High^ MuRCs, respectively, compared to 57 ± 23.7% in GM-FBS (mean ± SD, Fig. 5E).

**Fig. 6:**
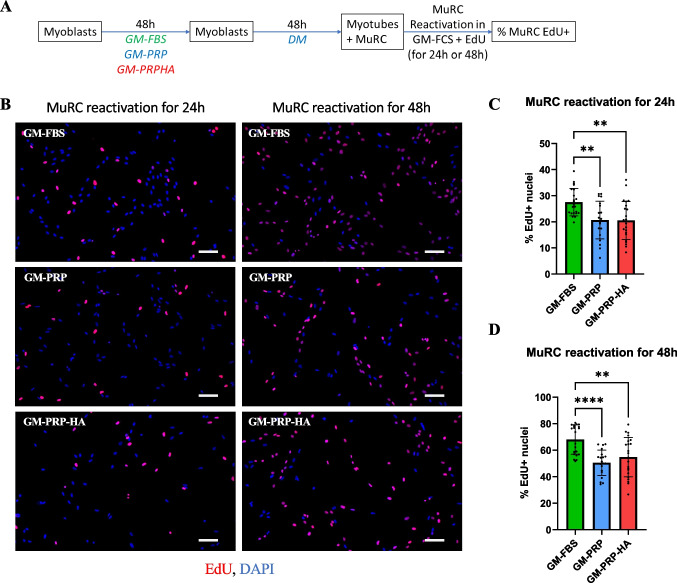
In vitro generated human MuRCs from myoblasts expanded in PRP need more time to re-enter the cell cycle. Human myoblasts were expanded for 48 h in GM-FBS, GM-PRP or GM-PRP-HA and switched to DM for 48 h. Differentiated cultures containing myotubes and MuRCs were then switched to GM-FBS containing EdU for 24 h or 48 h (A). (B) Representative images after 24 h or 48 h of re-activation, with EdU staining (red) and DAPI (blue) (Scale bar = 100 µm). (C) (D) Quantification of EdU^+^ cells after 24 h or 48 h of reactivation in GM-FBS (N = 20–24 pictures per condition, means ± SD, Statistics were calculated with a Mann–Whitney test (C) or Kruskal–Wallis test (D) and Dunn’s multiple comparisons test. Data are expressed as means ± SD. *p < 0.05, **p < 0.01, ***p < 0.001, ****p < 0.0001)

We have previously shown that Pax7^high^ MuRCs exhibit characteristics of MuSCs in deeper quiescence with a delay in cell cycle re-entry after re-activation [8]. Therefore, we evaluated the kinetics of cell cycle re-entry in MuRCs using EdU incorporation (Fig. 6A). We observed that MuRCs generated from GM-PRP or GM-PRP-HA myoblasts required more time to re-enter the cell cycle compared to MuRCs generated from GM-FBS myoblasts (Fig. 6B). After 24 h of re-activation, we showed that the percentage of EdU^+^ MuRCs was decreased in the PRP group, with 20.7 ± 7.2% and 20.5 ± 7.3%, respectively for GM-PRP and GM-PRP-HA as compared to 27.6 ± 5.3% for GM-FBS (mean ± SD, Fig. 6C). Similarly, we observed that the percentage of EdU^+^ MuRCs was also decreased after 48 h of reactivation in GM-PRP or GM-PRP-HA, with respectively 50.5 ± 9.6% and 54.8 ± 14.8% of Edu^+^ cells as compared to 68.0 ± 11.1% in GM-FBS (mean ± SD, Fig. 6D).

## Discussion

Human muscle reserve cells (MuRCs) have emerged as a promising source of stem cells for potential therapeutic applications in skeletal muscle diseases [7, 8]. However, the use of xenogeneic products such as fetal bovine serum (FBS), as a supplement to growth culture media, raises numerous safety and regulatory concerns that must be addressed prior to the production of human MuRCs batches for potential clinical applications [11–13]. Human serum and human PRP derivatives were tested as potential FBS substitutes, which effectively promoted the in vitro proliferation of human mesenchymal stem cells (MSC) [51–53] and of human fibroblasts [33]. To date, only a few studies have evaluated the viability of human sera and platelet derivatives in myogenic cell cultures, with conflicting results reported [38, 39, 54] [40]. Furthermore, although an autologous blood product may be ideal for therapeutic use, the limited availability of such autologous products underlines the need to explore the use of allogeneic blood products.

In the present study, we investigated whether non-activated allogeneic PRP or PRP-HA can efficiently replace xenogeneic FBS in the ex vivo expansion and differentiation of human primary myoblasts. We first showed that replacement of FBS by either fresh human allogeneic PRP or fresh human allogeneic PRP-HA, results in a similar proliferation rate with no significant increase in cell mortality. These results underline the pro-proliferative effects of these two nutritive supplements (PRP and PRP-HA) and suggest that allogeneic PRP represent a viable alternative to xenogeneic FBS for the in vitro expansion of human primary myoblasts. The exact composition of growth factors (GFs) in the PRP batches used in our study was not quantified. Nevertheless, it was shown that freshly human PRP preparations contain at least the following GFs: IGF, TGF-β, bFGF, PDGF-AB, EGF and VEGF (unpublished confidential data) in accordance with previously published data in which the composition of various PRP preparations have been documented [30, 55]. We also observed that the expression of various cell surface markers including HLA-ABC, HLA-DR, or ICAM1, known to be overexpressed in inflammatory conditions, was maintained in human myoblasts expanded in either PRP or PRP-HA. Moreover, we obseverd that the level of CD56 expression was similar in FBS-myoblasts and in PRP-myoblasts. This finding is important for the advancement of cell therapies, as it indicates that allogeneic PRP can be used in culture medium without altering myoblast viability or inducing excessive inflammatory responses. This aspect takes on added significance as we have recently found that primary human myoblasts have immunomodulatory capabilities [10], highlighting the therapeutic potential of combining these two tools. Nevertheless, our observations also suggested reduced myogenic commitment capacity (fusion indexes) of human myoblasts expanded in either GM-PRP or GM-PRP-HA in agreement with a previous study [40]. It has been described that the addition of heparin to the culture medium increases the proliferation of MuSCs and decreases the subsequent differentiation of these cells [40]. However, in our in vitro model, we didn't observe that the addition of heparin reduced the proliferation and differentiation capacity of cultured human myoblasts. We also demonstrated that human MuRCs, generated from human myoblasts expanded in GM-PRP or GM-PRP-HA, express higher levels of the transcription factor Pax7, compared to MuRCs generated from myoblasts expanded in GM-FBS. These data are particularly interesting as we have recently reported that Pax7^high^ MuRCs are in a deeper quiescent state and may represent a potential cell source for future cell therapy applications [8]. To evaluate the quiescent state of human MuRCs, we analyzed the cell cycle re-entry kinetic of MuRCs and we observed that human MuRC-PRP, and to a greater extent human MuRC-PRP-HA, required more time to re-enter the cell cycle compared to control MuRC-FBS. These data demonstrate that human myoblasts expanded in GM-PRP or GM-PRP-HA allow the generation of human MuRCs expressing higher levels of Pax7, indicating, as observed in rat muscle [56], that PRP can increase Pax7 expression in myogenic stem cells. We also evaluated if PRP-HA is an appropriate xeno-free alternative to FBS in our in vitro model. PRP-HA consists of a mix of half PRP and half non-crosslinked HA. HA, one of the main component of the extracellular matrix, has recently been identified as important for the overall regenerative process [57], for wound healing [58], and as a regulator of inflammation and cell migration [59]. More recently, HA has also been described as a key player in the MuSC niche, allowing them to communicate properly with infiltrating immune cells [45]. In our in vitro model, we observed that human myoblasts expanded in GM-PRP-HA allowed the generation of human MuRCs expressing higher levels of Pax7, suggesting that HA may play a role in creating a conducive niche for MuRCs, promoting their transition into a deeper state of quiescence.

In conclusion, our study presents an alternative xeno-free approach to FBS and our data indicate that allogeneic PRP or PRP-HA are suitable for the expansion of human myoblasts prior their myogenic differentiation. Moreover, we observed that myoblasts expanded in allogeneic PRP gave rise to human MuRCs in a deeper quiescent state (Pax7^High^). This research represents a significant step forward in the pursuit of clinical-grade cell therapies for various musculoskeletal conditions. Further studies are warranted to explore the transcriptional signature of human MuRC, to decipher the specific molecular pathways affected by PRP and PRP-HA, and to determine how PRP pre-treatment of human myoblasts can promote the in vitro generation of Pax7^High^ MuRCs.

## Supplementary Information

Below is the link to the electronic supplementary material.

Supplementary file1 (DOCX 283 KB)

## Data Availability

The datasets used and/or analyzed during the current study are available from the corresponding author upon reasonable request.
