# Supplementary material for: Platelet-Rich Plasma Promotes the Expansion of Human Myoblasts and Favors the In Vitro Generation of Human Muscle Reserve Cells in a Deeper State of Quiescence
Source: Stem Cell Rev Rep. 2024 Jul 13;20(7):1981–94. doi: 10.1007/s12015-024-10760-0 (PMC11445347; doi:10.1007/s12015-024-10760-0)

**Supplementary Figure 1: Full-length blots of Figure 5A.**

Human myoblasts were expanded in GM-FBS, GM-PRP or GM-PRP-HA for 48 hours and then switched to DM for 48 hours. Human MuRCs were isolated and analyzed for Pax7 expression by Western blot. Representative Western blot for Pax7 and α-tubulin expression.


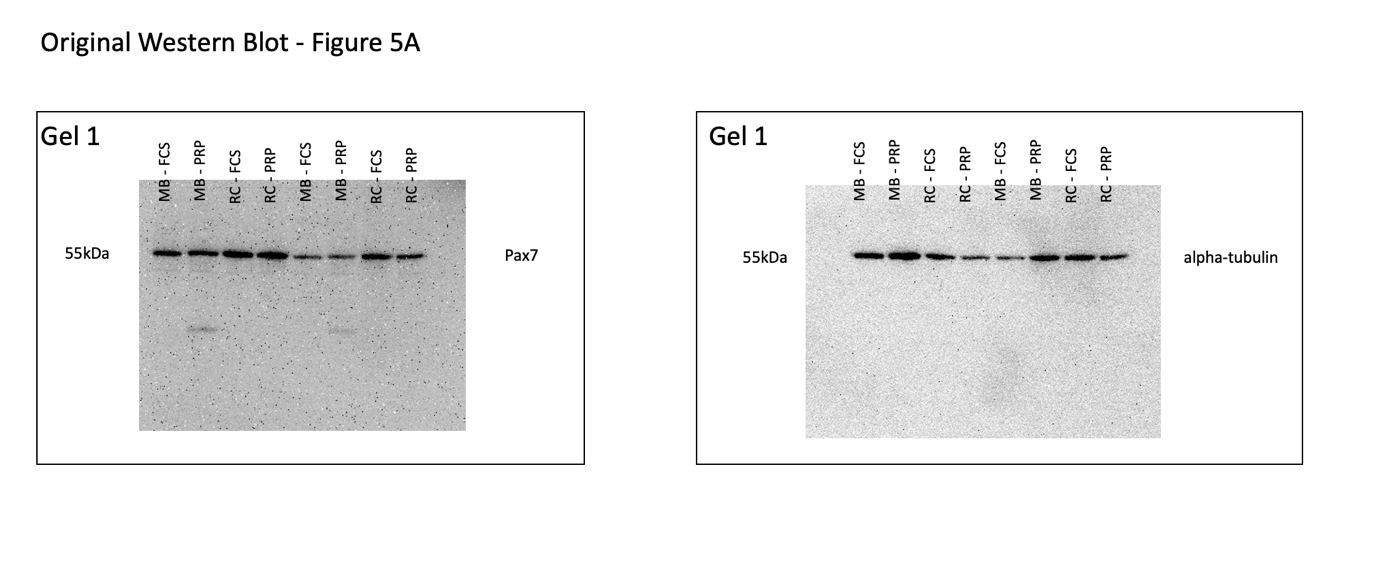

Supplement: Supplementary file 1 — Supplementary file1 (DOCX 283 KB) [file 12015_2024_10760_MOESM1_ESM.docx]
